# Nematic topological defects positionally controlled by geometry and external fields

**DOI:** 10.3762/bjnano.9.13

**Published:** 2018-01-10

**Authors:** Pavlo Kurioz, Marko Kralj, Bryce S Murray, Charles Rosenblatt, Samo Kralj

**Affiliations:** 1 Jozef Stefan International Postgraduate School, Jamova 39 , 1000 Ljubljana, Slovenia; 2 Comtron, Trzaska 21, 2000 Maribor, Slovenia; 3 Department of Physics, Case Western Reserve University, Cleveland, Ohio 44106-7079, USA; 4 Department of Physics, Faculty of Natural Sciences and Mathematics, University of Maribor, Koroska cesta 160, 2000 Maribor, Slovenia; 5 Jozef Stefan Institute, Jamova 39, 1000 Ljubljana, Slovenia

**Keywords:** nanoparticles, nematic liquid crystals, topological charge, topological defects

## Abstract

Using a Landau–de Gennes approach, we study the impact of confinement topology, geometry and external fields on the spatial positioning of nematic topological defects (TDs). In quasi two-dimensional systems we demonstrate that a confinement-enforced total topological charge of *m* > 1/2 decays into elementary TDs bearing a charge of *m* = 1/2. These assemble close to the bounding substrate to enable essentially bulk-like uniform nematic ordering in the central part of a system. This effect is reminiscent of the Faraday cavity phenomenon in electrostatics. We observe that in certain confinement geometries, varying the correlation length size of the order parameter could trigger a global rotation of an assembly of TDs. Finally, we show that an external electric field could be used to drag the boojum fingertip towards the interior of the confinement cell. Assemblies of TDs could be exploited as traps for appropriate nanoparticles, opening several opportunities for the development of functional nanodevices.

## Introduction

Topological defects (TDs) [1] represent an interdisciplinary research area [2] that is of high interest for nearly all branches of science. Due to their topological origin they exhibit several universal features that are independent of the microscopic details of the system in which the TDs appear. Their complete understanding might even resolve some of the most intriguing unanswered questions of nature: There are several strong indications that fields represent the basic entities of nature and not fundamental particles [3], which are, in this case, an emergent phenomenon. For example, as far back as 1962, Skyrme [4] developed a theory in which he described hadrons as topological defects in the pion field.

A convenient system in which to study the fundamental behavior of TDs are various liquid crystal (LC) phases [5]. They are relatively easily accessible to various experimental methods [6] due to their unique combination of optical anisotropy and transparency, fluid character, and mechanical softness. In addition, the diversity of LC phases and structures guarantees the existence of many qualitatively different TD structures.

Several recent studies reveal that TDs in LCs could efficiently control the position of assemblies of appropriate nanoparticles (NPs) [7–9]. Note that order parameters, which can host defects, possess two qualitatively different components [10]: an amplitude (also referred to as a hydrodynamic) field, and a symmetry breaking (also referred to as a gauge or nonhydrodynamic) field. If the characteristic size of a nanoparticle is comparable to the amplitude correlation length of an order parameter (which roughly estimates the core size of a defect), and if the nanoparticle does not sufficiently disturb the symmetry breaking field surrounding the core of the defect, then the defect could efficiently trap the NP due to the defect core displacement (DCR) mechanism [11]. In this case, a relatively energetically expensive defect core volume is (at least partially) replaced by the volume of the NP, thereby reducing the overall energy. It has been shown that lattices of orientational (disclinations) [11–12] and translational (dislocations) [9] defects can readily trap such NPs. Furthermore, it was demonstrated that line defects could be exploited to form nanowire-type structures [9,13] consisting of NPs.

In this contribution we study numerically the effects of geometry and an external electric field on the positions of nematic TDs using the Landau–de Gennes mesoscopic approach.

## Theoretical Background

Of interest is the impact of confinement and/or an external electric field on topological defects in a nematic liquid crystal. We use the Landau–de Gennes approach [5] in terms of the tensor order parameter 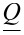. In its eigenframe it is expressed as 
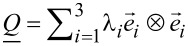
, where 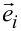 and λ*_i_* are the corresponding eigenvectors and eigenvalues, respectively. We consider uniaxial LCs where the bulk equilibrium ordering is described by the uniaxial tensor

[1]
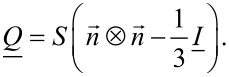


The unit vector 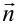 points along the local uniaxial direction and is referred to as the nematic director. The uniaxial orientational order parameter, *S*, quantifies the extent of fluctuations along 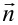, and 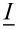 is the unit tensor. If the LC ordering is distorted, the system can exhibit biaxial states. In simulations we study topological defects either in the Cartesian coordinate system (*x,y,z*) or in the cylindrical coordinate system (*r*,φ,*z*). Their coordinate frames are determined by the unit vectors 
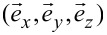
 and 
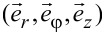
, respectively. We parameterize the nematic order parameter as

[2]
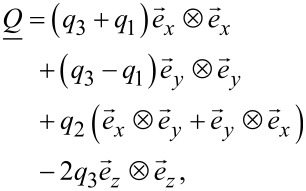


[3]
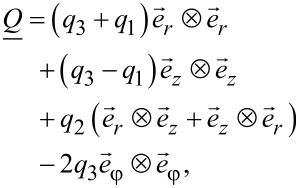


where {*q*_1_,*q*_2_,*q*_3_} are the variational parameters.

A convenient metric for the degree of biaxiality is the biaxiality parameter [14–15]:

[4]
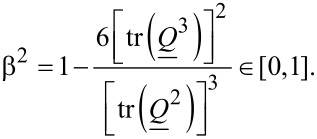


A uniaxial state and configurations with maximum biaxiality are denominated by β^2^ = 0 and β^2^ = 1, respectively.

### Free energy and scaling

We write the free energy as the sum of volume and surface integrals:


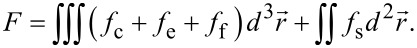


The condensation (*f*_c_), elastic (*f*_e_) and external electric field (*f*_f_) free energy densities are expressed as [5,15]:

[5]
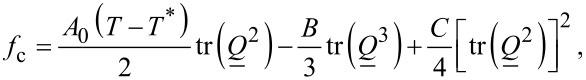


[6]
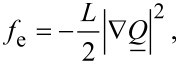


[7]
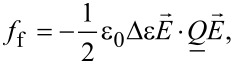


respectively. The quantities *A*_0_, *B*, *C* are material constants, T* is the supercooling temperature, *L* is the representative characteristic elastic constant in the single elastic constant approximation, 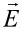 is the external electric field, ε_0_ is the permittivity of free space, and Δε is the dielectric constant anisotropy. We model conditions at the LC confining boundaries either by [15–16]:

[8]
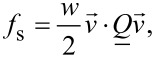


or

[9]
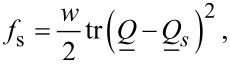


where *w* is the surface interaction strength, 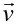 is the surface normal of the local confinement, and 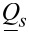 describes preferred nematic ordering of the surface. The surface term given by Equation 8 enforces for *w* > 0 (*w* < 0) degenerate tangential (homeotropic) anchoring. On the other hand, the contribution in Equation 9 is minimized for 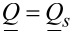, assuming *w* > 0. In simulations we consider cases where 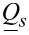 enforces uniaxial ordering given by Equation 1, where *S* possesses the bulk equilibrium value, and [5,17]:

[10]
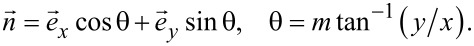


This ansatz enforces a topological defect of strength *m*, where *m* is an integer multiple of 1/2.

For numerical and presentational convenience [15], we introduce the reduced temperature


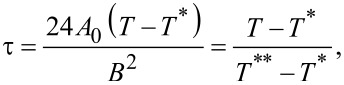


where T** is the nematic superheating temperature. In this scaling the bulk phase transition temperature *T_IN_* corresponds to


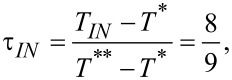


and the bulk degree of uniaxial ordering minimizing Equation 5 can be expressed as


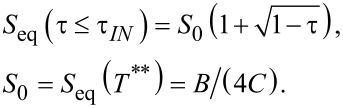


The materials properties of the LC are reflected in various characteristic lengths describing the responses of LC ordering to different perturbations. The relevant lengths for our study are the biaxial correlation length, ξ_b_, the external field coherence length, ξ_E,_ and the surface extrapolation length *d*_e._ We define them as follows:

[11]
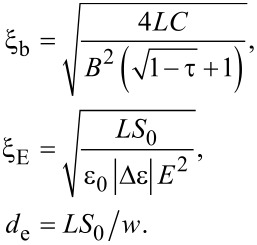


Note that for scaling purposes we express the latter two distances at *T* = T**. To estimate their values we choose materials properties of a typical nematic LC, 4-cyano-4'-pentylbiphenyl (5CB), for which [5] *A*_0_ ≈ 0.3·10^0^ J/(K·m^3^), *B* ≈ 4.8·10^6^ J/m^3^, *C* ≈ 1.6·10^7^ J/m^3^, *L* ≈ 10^−11^ J/m, Δε ≈ 5, *T_IN_* ≈ 318 K, *T_IN_* − T* ≈ 1.1 K, T** − *T_IN_* ≈ 0.18. For temperatures close below *T_IN_* it follows ξ_b_ ≈ 20 nm, ξ_E_(*E* ≈ 10^6^ V/m) ≈ 1 μm, *d*_e_(*w* ≈ 10^−4^ J/m^2^) ≈ 1 μm.

We obtained nematic structures for the given boundary conditions by minimizing the total free energy of the system. The resulting Euler–Lagrange equilibrium equations for the variational parameters {*q*_1_,*q*_2_,*q*_3_} are solved using the standard over-relaxation method, the calculation details of which are given in [15].

### Geometry of the problem

**Figure 1:**
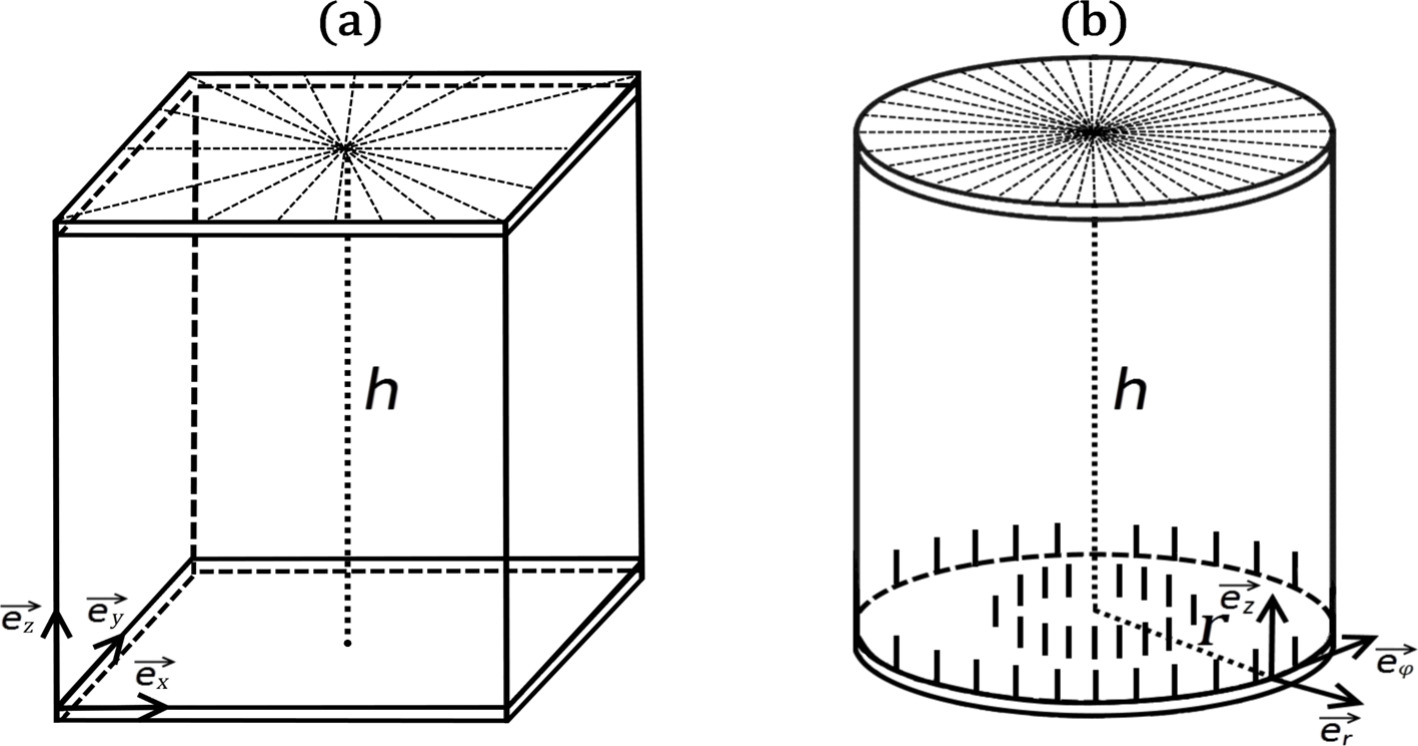
Geometry of cells used in simulations. (a) In the “Cartesian” cells we enforce at the top “master” plate uniaxial nematic structures defined by Equation 10 and assume 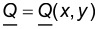. (b) In the “cylindrical” cells we impose a boojum topological defect at the top plate and assume 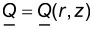.

We consider thin plane-parallel cells of thickness *h.* The top and bottom plates are placed at *z* = 0 and *z* = *h*, respectively. We consider the cells either in the Cartesian (*x,y,z*) or cylindrical geometry (*r*,φ,*z)*, as illustrated in Figure 1. Accordingly, we use two different boundary conditions, to which we refer as “boundary anchoring condition” (BAC) [18] and “surface anchoring condition” (SAC), respectively.

We use BAC in the “Cartesian” cells. We assume that the cells are relatively thin and the nematic ordering is entirely dominated by conditions at the top “master” plate. At the master plate we impose a strong uniaxial boundary condition of order parameter 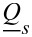 qiven by Equation 10. The latter is either a circle of radius 
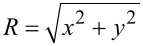
 or a trapezoid that is characterized by a distance *R*. It is assumed that *R* is large with respect to the relevant nematic order correlation length. Inside the boundary we allow the nematic tensor frame to freely rotate in the (*x,y*)-plane. In these simulations the nematic ordering is effectively two-dimensional. Hence, we neglect variations along the *z*-axis and set 
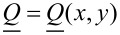
.

Regarding SAC we consider thicker cells and permit spatial variations along the *z*-direction. We perform simulations in the cylindrical coordinate system [19] and we impose the cylindrical symmetry, 
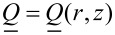
. At the top plate we enforce uniaxial boundary conditions 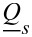 given by Equation 10, in which we set *m* = 1. At the bottom plate we enforce homeotropic anchoring conditions using the ansatz in Equation 8. At the lateral boundaries we assume free boundary conditions. These conditions impose a boojum topological defect at the top plate [19–20].

**Figure 2:**
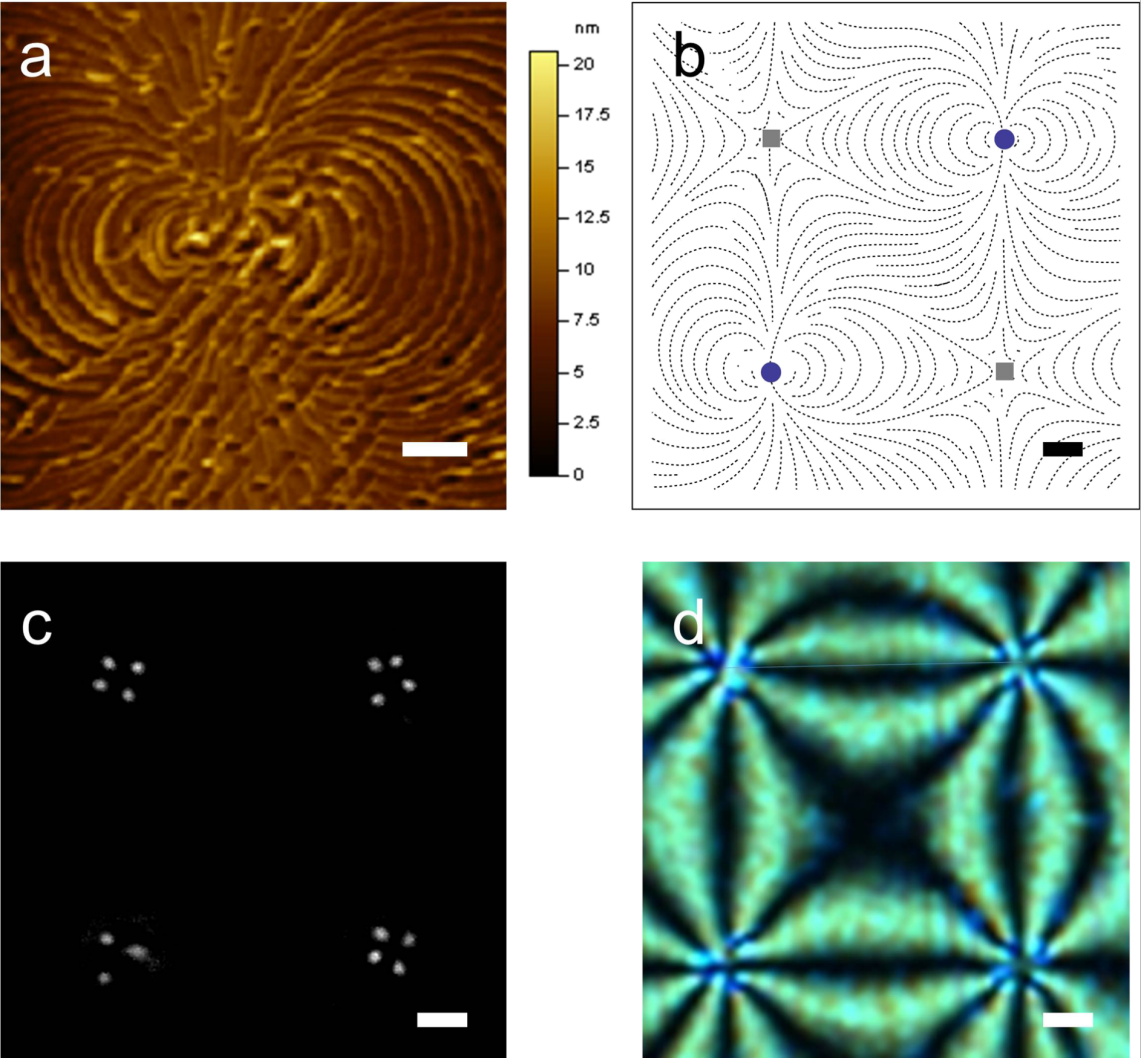
(a) Typical scribed surface topography enforcing the *m* = 2 topological defect. (b) Schematic representation showing the AFM-scribed director pattern of four topological defects (*m* = 2: blue circles, *m* = −2: gray squares), part of a larger square array of such defects. The separation between neighboring defects is roughly 30 μm, *h* ≈ 3 μm. (c) Darkfield microscopy image of a nematic cell the master plate of which enforces a square array of *m* = ±2 TDs; this image shows a rare example of a double integer defect that decomposes into a pair of half integer defects plus one integer defect. (d) Typical polarizing microscopy pattern image of this defect array. The scale bars are (a) 500 nm and (b,c,d) 5 μm.

Note that in our simulations we mimic geometric set-ups that could be realized experimentally using, for instance, the atomic force microscope (AFM) scribing method [17]. In a typical experimental set up one confines a nematic LC within a thin plane-parallel cell, where at least one (“master”) surface imposes anchoring conditions inscribed via an AFM stylus [17–18], with a planar degenerate “slave” as the other surface. In Figure 2 we depict an example of a ”master” substrate enforcing 2D topological defects of strength *m* = ±2, and the corresponding experimentally measured textures using polarized optical microscopy.

## Results and Discussion

In the following we present results of our simulations. We consider structures using the “Cartesian” cells, where we study how different patterns of TDs emerge. Afterwards we focus on an external field defect core structure driven changes in the “cylindrical” cell.

### Faraday cavity effect

**Figure 3:**
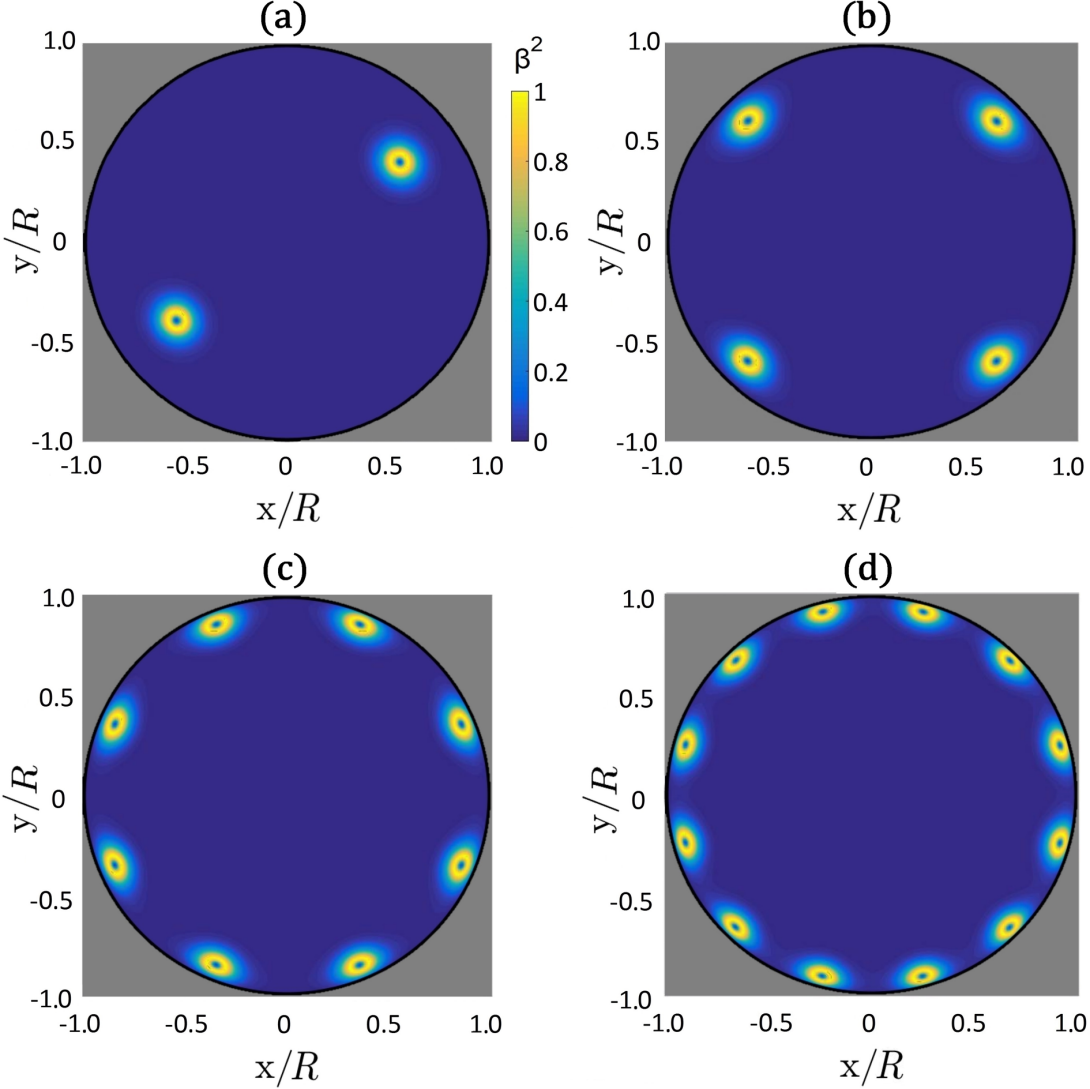
Plots of β^2^(*x*,*y*) for different imposed total topological charges using BAC: (a) *m* = 1, (b) *m* = 2, (c) *m* = 4, (d) *m* = 6. TDs are decomposed into elementary units bearing the charge *m*_0_ = 1/2, which assemble close to the bounding circle. In all panels we set R/ξ_b_ = 30 and τ = −8. The corresponding director field is depicted in Figure 4.

**Figure 4:**
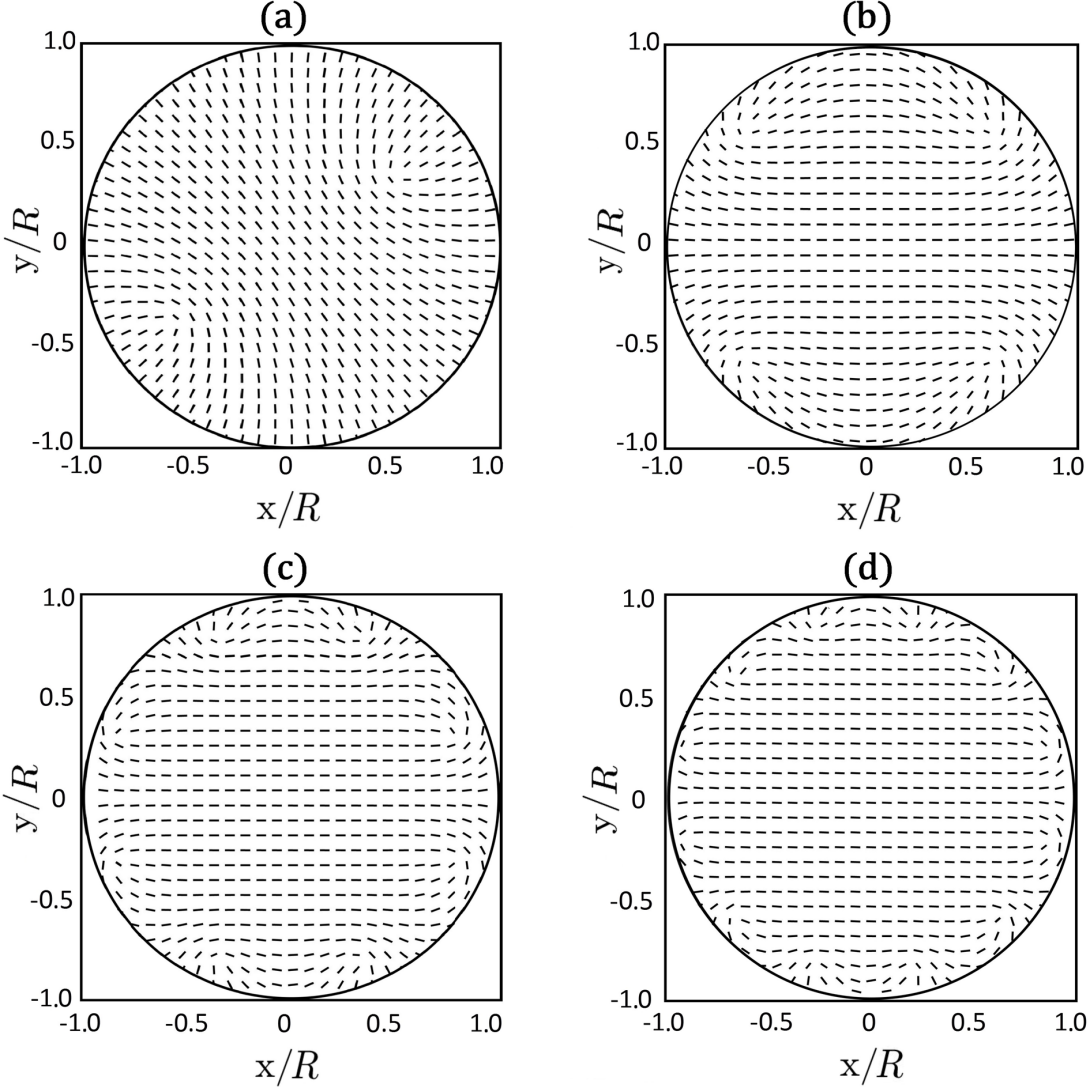
2D Plot of the eigenvectors of 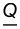 with the largest positive eigenvalue, corresponding to the 2D biaxiality profiles β^2^(*x*,*y*) plotted in Figure 3. (a) *m* = 1, (b) *m* = 2, (c) *m* = 4, (d) *m* = 6. In all panels we set R/ξ_b_ = 30 and τ = −8.

We first study patterns emerging from the BAC boundary condition. We enforce a total topological charge of strength *m* inside the circular boundary of radius *R*. At the boundary we strongly impose the nematic ordering defined by Equation 10. The energy-minimized configurations are plotted in Figure 3 and Figure 4.

In Figure 3 the equilibrium biaxiality profiles are plotted, in which cores of TDs are clearly visible. The imposed total charges always decompose into TDs bearing elementary charges *m*_0_ = 1/2. The fingerprint of the cores of *m*_0_ = 1/2 TDs is a volcano-like rim where β^2^ = 1 [15,18]. For *m* = 1, *m* = 2, *m* = 4 and *m* = 6 the patterns exhibit 2, 4, 8 and 12 TDs, respectively. The TDs tend to assemble close to the boundary. The resulting director orientation is plotted in Figure 4: The eigenvectors of 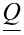 with the largest positive eigenvalue (which we set to be 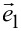) are plotted. These correspond in the uniaxial limit to the nematic director field. Note that nematic textures are essentially uniaxial, except close to the defects cores. For this reason we henceforth refer to 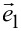 as the nematic director field. The ordering becomes increasingly spatially uniform in the central region with increasing values of *m*.

This phenomenon is reminiscent of the Faraday cavity effect in conductors. Namely, if one puts electric charges on a conducting body, the charges assemble at its surface and the resulting electric field inside the body vanishes. The Faraday-like behavior in our simulations is clearly visible for cases of *R*/ξ_b_ >> 1. In our simulations, we set *R*/ξ_b_ = 30, and for typical LCs ξ_b_ is approximately 20 nm. The absence of an electric field inside the conductor in the electrostatic analogue corresponds in our simulations to a spatially uniform nematic director in the area separated by a distance greater than ξ_b_ from the confining boundary.

### Temperature-induced pattern changes

**Figure 5:**
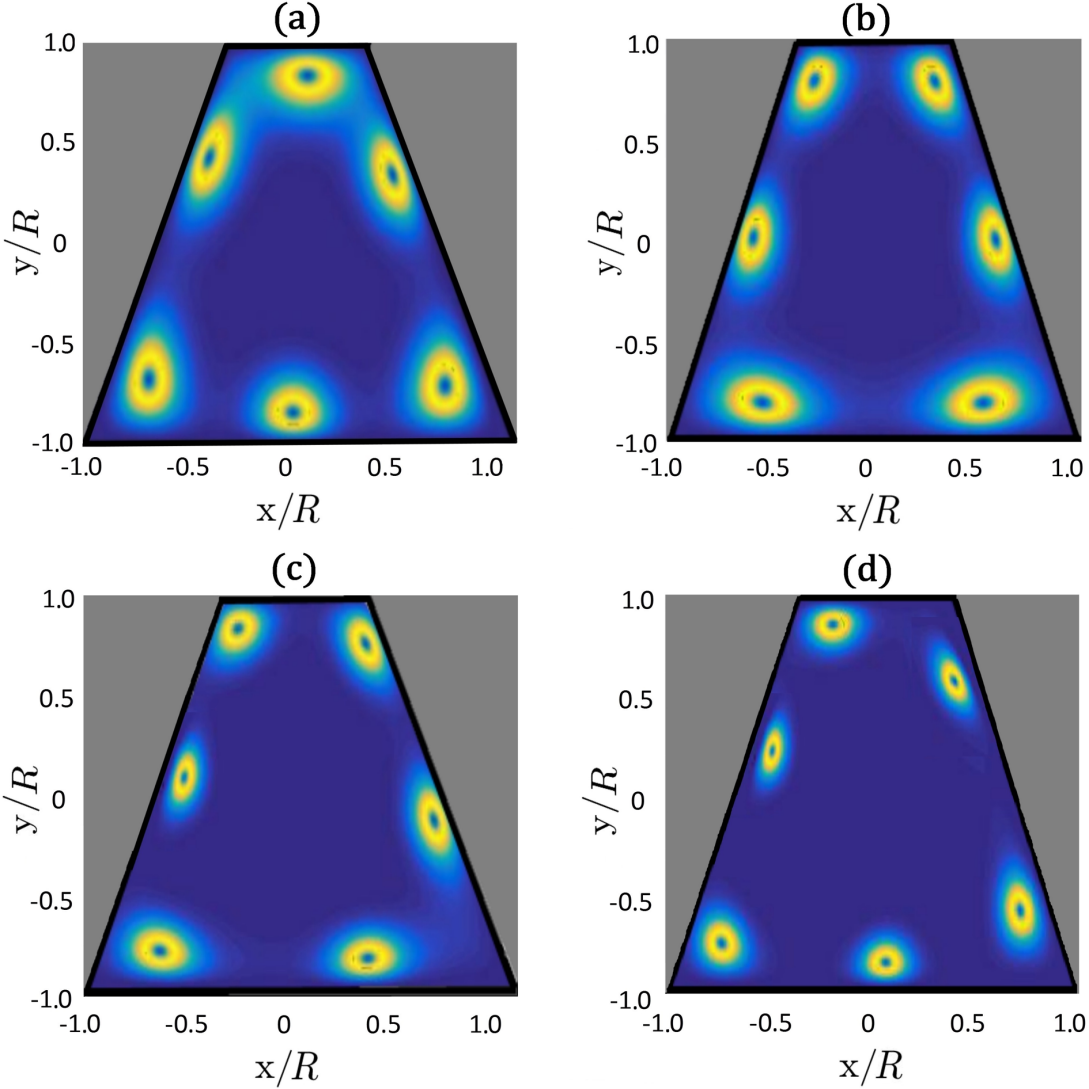
Plots of β^2^(*x*,*y*) of the configuration of TDs with increasing ratio η = *R*/ξ_b_: (a) η = 14, (b) η = 17, (c) η = 21, (d) η = 25. In practice this could be achieved by decreasing the temperature of the sample. The trapezoid boundary enforces a total topological charge of *m* = 3, which splits into six elementary charges *m*_0_ = 1/2.

**Figure 6:**
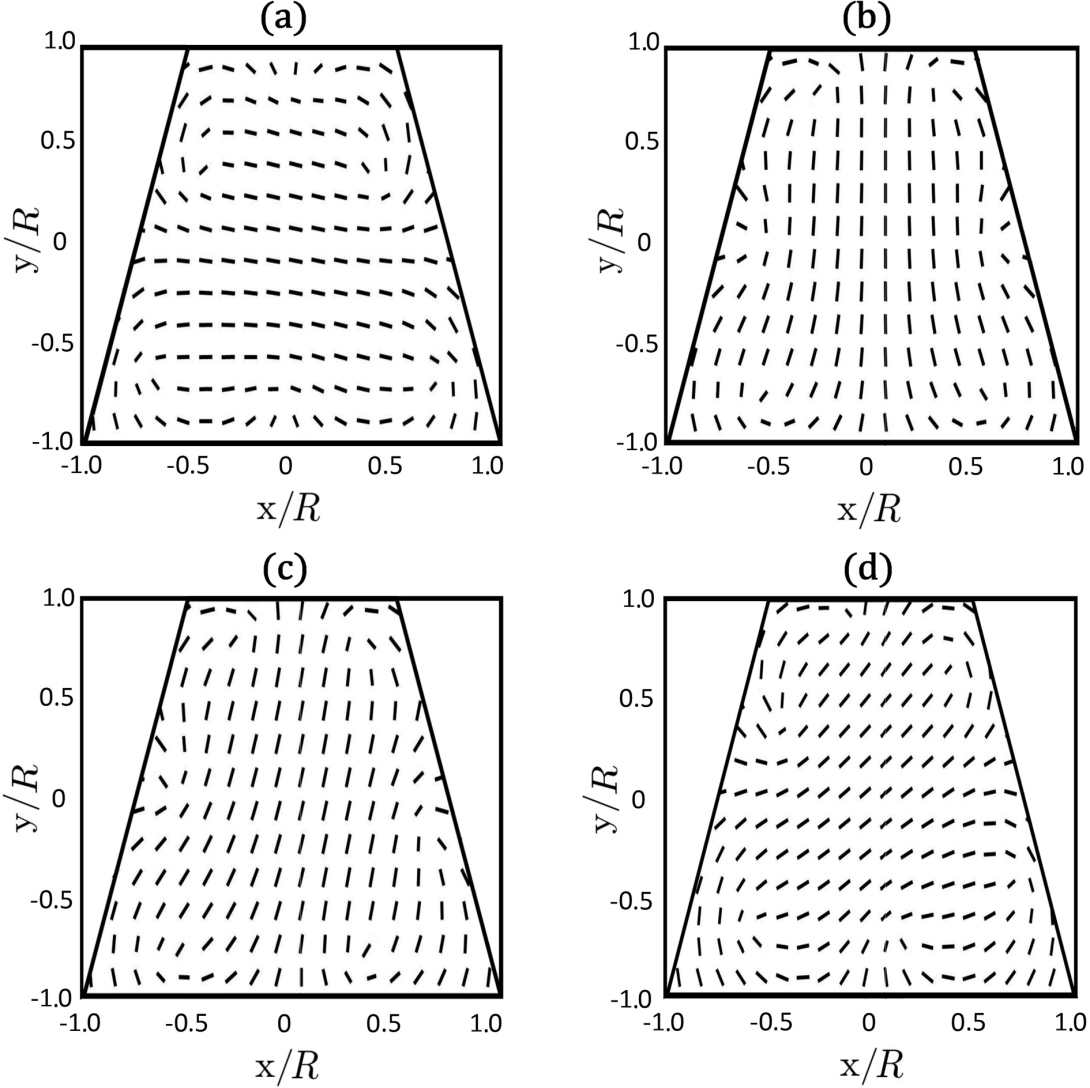
Changes in the nematic director field with increasing ratio η = *R*/ξ_b_: (a) η = 14, (b) η = 17, (c) η = 21, (d) η = 25. The corresponding biaxiality profiles are depicted in Figure 5.

We next consider cases in which we change the symmetry of the bounding surface. In Figure 5 and Figure 6 we show nematic configurations for a trapezoid-shaped boundary, through which we enforce *m* = 3 using Equation 10. In all cases the imposed charge decays into elementary charges *m*_0_ = 1/2*,* and the charges assemble at the confining boundary as discussed in the previous subsection. For this specific confinement symmetry we observe changes in the nematic patterns when the ratio η = *R*/ξ_b_ is decreased, where *R* corresponds to the bottom length of the trapezoid.

In practice, η can be varied by changing temperature of the sample, which affects ξ_b_. In this case, a rotation of defect patterns is expected according to our simulations. The changes in patterns reveal that the energy landscape of the system substantially changes with varying η. From the perspective of TDs, the LC configurations reflect the interplay between mutual repulsion among defects and interaction of TDs with the confinement geometry. Note that for sufficiently symmetric confinements, the “rotation” disappears. It is also sensitive to the number of TDs.

### A boojum tip driven by an external field

**Figure 7:**
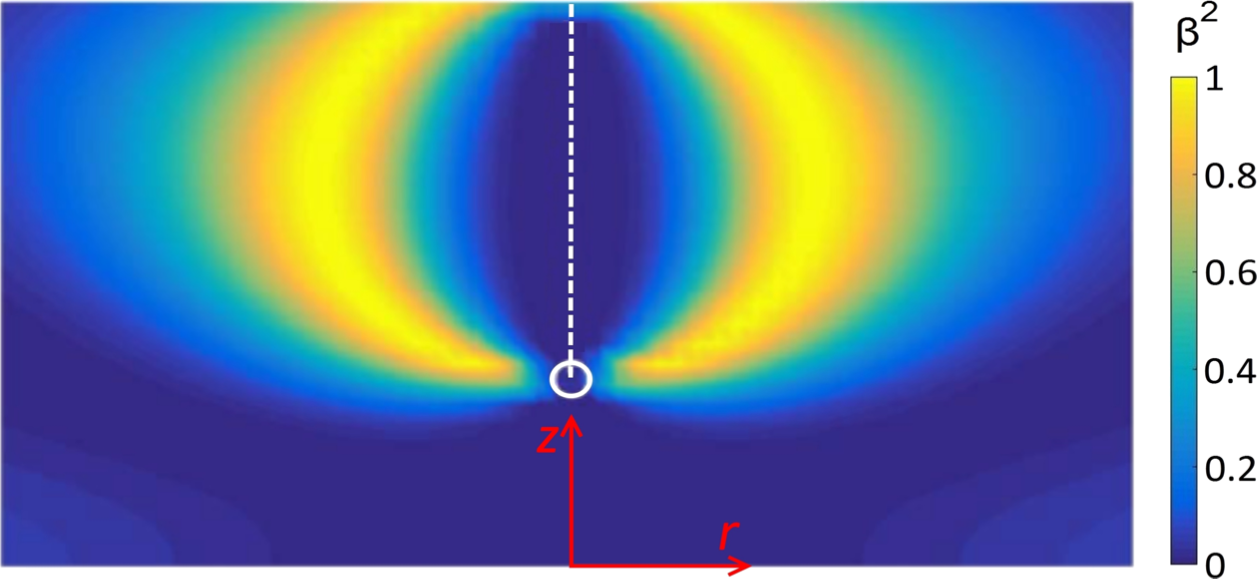
Cross section through a cylindrically symmetric boojum core structure. The biaxial shell exhibiting maximal biaxiality joins the melted fingertip with the top plate. In the given case, the anchoring strength at the top plate is finite. The negative uniaxial region is indicated by a dashed white line. The fingertip is marked with a circle. The color bar indicates the values of β^2^.

**Figure 8:**
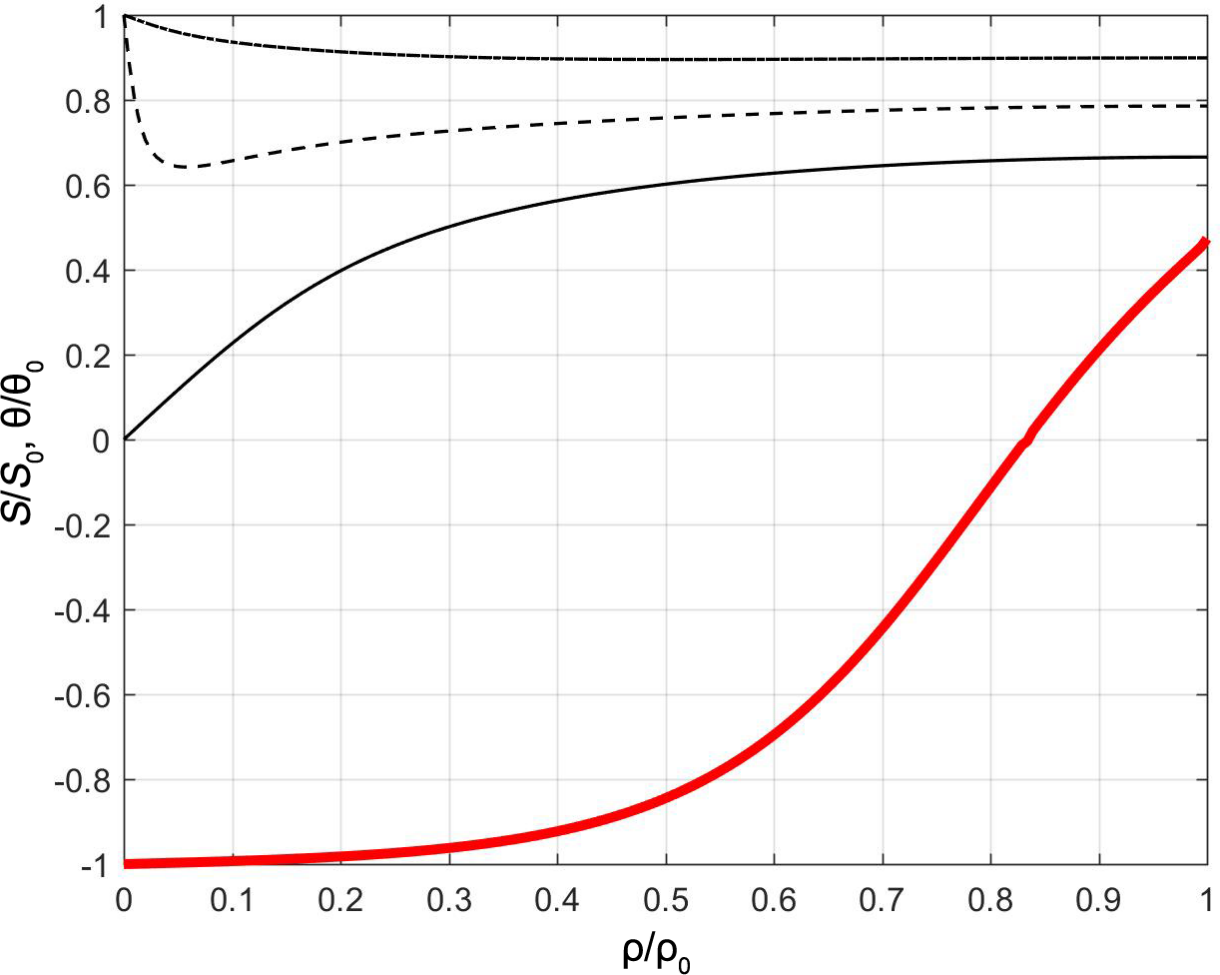
Radial spatial variation of the director field (thin black lines, ρ ≡ *r*, ρ_0_ ≡ ξ_b_; θ_0_ = π/2), and degree of uniaxial order along the symmetry axis (thick red line, *z* ≡ ρ, ρ_0_ ≡ *h*; *S*_0_ = |*S*(*r* = 0, *z* = *h*)|, r = 0). In the simulations we establish a strong anchoring condition at the top plate, *h*/ξ_b_ = 8, τ = −8.

We next consider thicker cells and SAC boundary conditions. For sufficiently strong anchoring, a boojum surface defect [19–20] occurs at the top surface. Its structure has been studied in detail in [19]. Its surrounding nematic director field resembles a “classical” half-hedgehog structure. The core structure is relatively complex [19] and is schematically shown in Figure 7. Its core is characterized by a negatively uniaxial finger, surrounded by a shell exhibiting maximal biaxiality, β^2^ = 1. The fingertip is melted due to the topology of the surroundings. To understand this let us consider ideal cylindrically symmetry, which we also adopt in our simulations. The symmetry axis of the defect is uniaxial. Namely, in terms of the parametrization defined by Equation 3 the elastic free energy density includes a term that is linearly proportional with [(3*q*_1_ + *q*_2_)^2^ + *q*_3_^3^]/*r*^2^. The singularity at *r* = 0 can be avoided if uniaxial states are introduced, for which 3*q*_1_ + *q*_2_ = *q*_3_ =0. When decreasing the value of *z* from the top plate, the negative uniaxiality extends to the fingertip (Figure 8). Below the tip the axis is positively uniaxial. The transition from negative to positive uniaxiality requires melting of the nematic ordering in the transition area. A typical spatial variation of the director field, 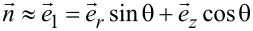, in the radial direction and order changes of the parameter along the symmetry axis are depicted in Figure 8. In the given case, the fingertip (where the nematic ordering is melted) is located at (*r*,*z*) = (0,*h* − ξ_f_), where ξ_f_ ≈ 0.8ξ_b_. For *z* > *h* − ξ_f_ the director field is roughly radial everywhere and *S*(*r* = 0,*z*) < 0. For *z* < *h* − ξ_f_ the nematic order is positively uniaxial at *r* = 0 and 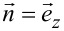. With increasing *r* the director field monotonically increases its departure from the z-axis.

**Figure 9:**
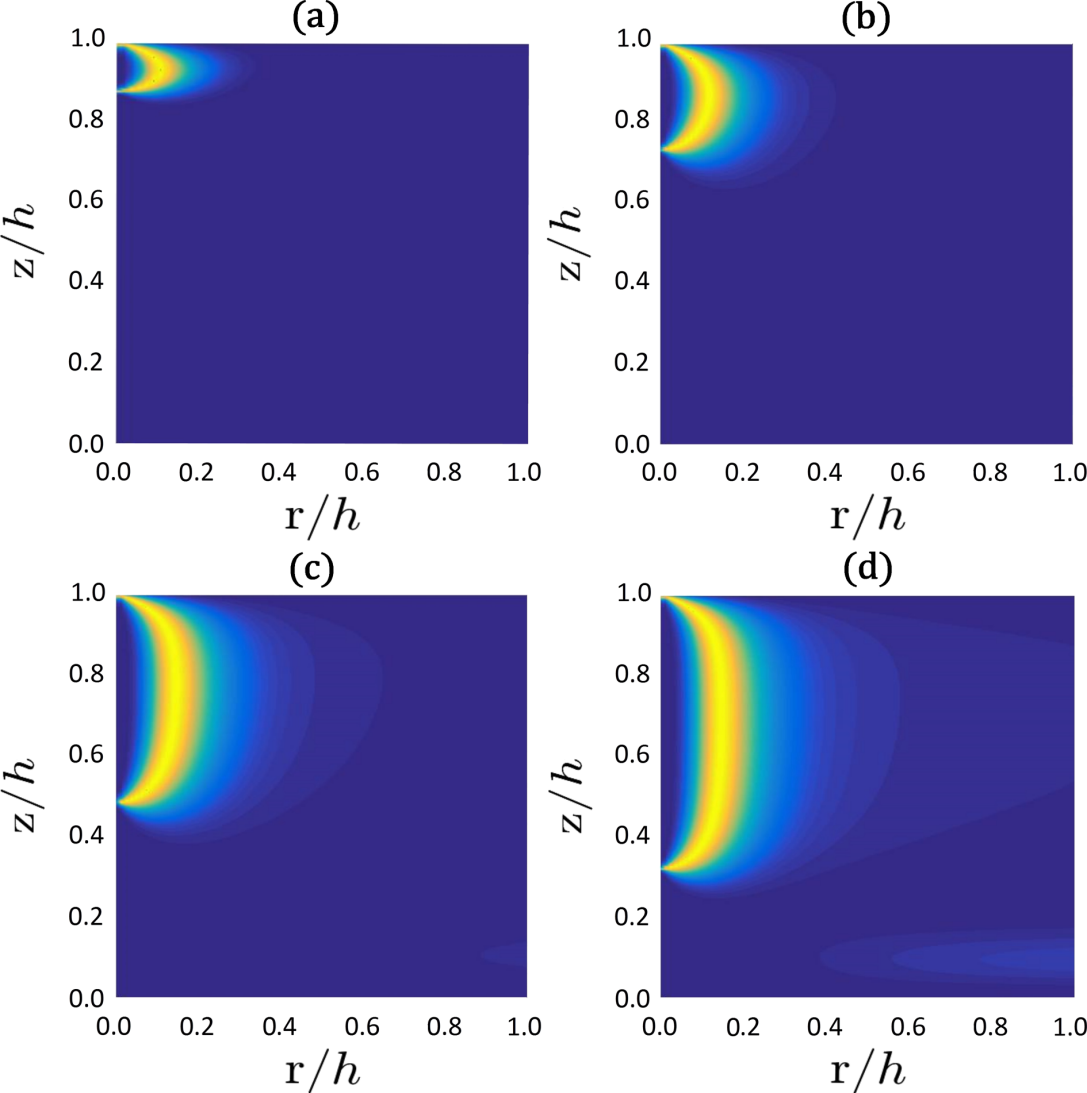
Extension of the fingertip with an increasing external field. Because of the cylindrical symmetry, we plot only the right-hand part of the boojum core structure (see Figure 8): (a) *h*/ξ_E_ = 0, (b) *h*/ξ_E_ = 8, (c) *h*/ξ_E_ = 10, (d) *h*/ξ_E_ = 12; *h* = 10ξ_b_, *h*/*d*_e_ = 100, τ = −8.

We next apply an external electric field along the *z*-axis and assume that the LC possesses a negative dielectric anisotropy (Δε < 0). In this case the external field favors the negatively uniaxial part of the boojum. Consequently, it becomes elongated on increasing the field strength, as it is illustrated in Figure 9. To achieve this, the external field must be relatively strong, i.e., ξ_E_ ≈ ξ_b_, which in a typical LC would correspond to *E* ≈ 10^8^ V/m.

## Conclusion

We studied numerically the impact of geometry, topology, and external fields on patterns and positions of nematic topological defects. In our phenomenological study we used the Landau–de Gennes approach in terms of the nematic tensor order parameter. We found that, in quasi 2D systems, TDs rearrange relatively close to the boundary line, which topologically enforces their presence. Such assemblies of TDs enable the formation of essentially spatially uniform orientational ordering in the central part of the confined nematic. This effect is reminiscent of the Faraday cavity phenomenon in electrostatics. Furthermore, we demonstrated that, for certain confinement geometries (we chose a trapezoid), one could induce collective rotation of a pattern of TDs on changing the defect core size. For example, the latter could be varied by changing the temperature. Furthermore, we demonstrated that one could extend the boojum finger towards the cell interior if an external field is imposed approximately along its symmetry axis for LCs exhibiting negative external field anisotropy.

These mechanisms could be exploited for indirect positional manipulation [7–9,11–12] of certain NPs via positionally controlled TDs. Namely, appropriately surface-decorated NPs could be efficiently trapped within cores of TDs through the defect core displacement mechanism. Therefore, by manipulating the texture of TDs, one could control or reconfigure the positions of trapped NPs, which could be exploited in future nano-scale devices.
